# Effect of Nesfatin-1 on Rat Humerus Mechanical Properties under Quasi-Static and Impact Loading Conditions

**DOI:** 10.3390/ma15010333

**Published:** 2022-01-03

**Authors:** Anna Skic, Iwona Puzio, Grzegorz Tymicki, Paweł Kołodziej, Marta Pawłowska-Olszewska, Kamil Skic, Karolina Beer-Lech, Marek Bieńko, Krzysztof Gołacki

**Affiliations:** 1 Department of Mechanical Engineering and Automation, Faculty of Production Engineering, University of Life Sciences in Lublin, 20-612 Lublin, Poland; pawel.kolodziej@up.lublin.pl (P.K.); karolina.beer-lech@up.lublin.pl (K.B.-L.); krzysztof.golacki@up.lublin.pl (K.G.); 2 Department of Animal Physiology, Faculty of Veterinary Medicine, University of Life Sciences in Lublin, 20-950 Lublin, Poland; g.tymicki@wp.pl (G.T.); marta.pawlowska@up.lublin.pl (M.P.-O.); marek.bienko@up.lublin.pl (M.B.); 3 Institute of Agrophysics, Polish Academy of Sciences, 20-290 Lublin, Poland; k.skic@ipan.lublin.pl

**Keywords:** rat humerus, nesfatin-1, osteopenia, impact loading, quasi-static conditions, SEM analysis

## Abstract

The investigations on the response of bone tissue under different loading conditions are important from clinical and engineering points of view. In this paper, the influence of nesfatin-1 administration on rat humerus mechanical properties was analyzed. The classical three-point bending and impact tests were carried out for three rat bone groups: control (SHO), the humerus of animals under the conditions of established osteopenia (OVX), and bones of rats receiving nesfatin-1 after ovariectomy (NES). The experiments proved that the bone strength parameters measured under various mechanical loading conditions increased after the nesfatin-1 administration. The OVX bones were most susceptible to deformation and had the smallest fracture toughness. The SEM images of humerus fracture surface in this group showed that ovariectomized rats had a much looser bone structure compared to the SHO and NES females. Loosening of the bone structure was also confirmed by the densitometric and qualitative EDS analysis, showing a decrease in the OVX bones’ mineral content. The samples of the NES group were characterized by the largest values of maximum force obtained under both quasi-static and impact conditions. The energies absorbed during the impact and the critical energy for fracture (from the three-point bending test) were similar for the SHO and NES groups. Statistically significant differences were observed between the mean F_i max_ values of all analyzed sample groups. The obtained results suggest that the impact test was more sensitive than the classical quasi-static three-point bending one. Hence, F_i max_ could be used as a parameter to predict bone fracture toughness.

## 1. Introduction

Bones belong to the anisotropic materials having different properties depending on the analyzed direction. The mechanical strength of bones depends on many factors. One of them is the function that the bone performs in the body. It has a significant influence on its mass, structure as well as the volumetric distribution of individual fractions. The bone tissue strength is also influenced by the age of the individual, sex, and health [1]. In order to identify the mechanical properties of bone material, it is important to understand the mechanical properties of its component phases and the structural relationship between them at the various levels of hierarchical structural organization [2]. At the macrostructure level, bone is distinguished into the cortical (compact) and cancellous (trabecular) types. The microstructure of cortical bone is composed of regular, cylindrically shaped lamellae [1,2] while the cancellous bone is made up of a series of interconnecting trabeculae forming a complex three-dimensional architecture. The cancellous bone material is more metabolically active and more often remodeled than the cortical bone. Cortical bone is considered stiffer and capable of withstanding greater loads than cancellous bone, but it is also more fragile. Its mechanical properties are influenced greatly by the porosity, the mineralization level, and the organization of the extracellular matrix [2,3].

In the study of bone mechanical properties, various types of deformation are used: tensile, compression, torsion, bending, and shear. Under physiological conditions, the bone is most often affected by the forces of compression, stretching, and bending. However, a bone fracture usually occurs as a result of a sudden trauma, a fall or impact, in which the bone is subjected to transverse forces [4]. Classical methods of examining the mechanical properties of bones are based on the quasi-static three-point bending test [5]. The bending strength represents the greatest stress occurring in the material at the moment of fracture [1,6]. A measure of a mechanical property is the deformation that results from an applied force. Initially, the change in stress is linear; with a further increase in stress, nonlinear reversible strains are observed. After exceeding the elastic limit, the deformation becomes irreversible (plastic) and a further increase in stress leads to the material breaking.

Bone is not only an anisotropic but also a viscoelastic material. Its mechanical behaviors depend on the loading rate [4]. So far, few studies have been carried out on the impact test of whole bones. Prot et al. [7] and Laporte et al. [8] analyzed the mechanical properties of cancellous bovine bone samples under the dynamic compression, using high strain rates. The authors concluded that the bone response to compression revealed a foam-type behavior over the whole explored range of strain rates. Zhai et al. [4] analyzed the loading rate effect on the fracture toughness of porcine bone specimens and found that the fracture initiation of cortical bone decreased as the loading rate increased. The impact of free-falling mass was well described for plant tissues [9,10,11] or engineering materials [12,13]. With regard to bone, there are still few studies describing the mechanical parameters under the impact loading condition.

The most common disease affecting the mechanical properties of bones is osteoporosis. It is characterized by mass loss and microarchitectural disorders, leading to the weakening of the bone structure and resistance [14,15]. Osteoporosis is mainly caused by an imbalance between the osteoblast-mediated bone formation and the osteoclast-mediated bone resorption [16]. Ovariectomy is the model most often employed for mimicking postmenopausal osteoporosis [17,18]. It results in rapid, profound osteopenia and changes in the microstructure of trabecular and cortical bones. The ovary removal results in deficient estrogen production and is responsible for the imbalance in bone turnover, such that resorption exceeds formation [19]. In rats, osteopenia resulting from ovariectomy bears a strong resemblance to human osteopenia, in both its anatomical features and in the transitional and steady states of bone dynamics [20,21]. Many international societies, including the US Food and Drug Administration (FDA) and the European Medicines Agency (EMA), recommend a rat model for the study of bone characteristics under an osteoporotic condition [22,23].

Many medications have been developed so far for the treatment of osteoporosis [24,25]. In recent years, extensive research has been carried out on the various adipokines that have an effect on the skeletal system [26,27,28]. Nesfatin-1 derived from nucleobindin-2 (NUCB-2) is one of the adipokines that can affect bone metabolism, but the research in this area is limited [16]. As nesfatin-1 is a pleiotropic peptide naturally produced in the body, studying its effects on bone tissue can be very helpful in preventing and treating bone disorders. The results obtained by Puzio et al. [14] showed that nesfatin-1 exerts a protective effect on the bone tissue properties in rats in the conditions of developing osteopenia. The authors showed an increase in the femur mechanical parameters of ovariectomized rats receiving nesfatin-1 under the quasi-static loading condition [14]. These results indicate that nesfatin-1 can be used in the prevention of bone loss that leads to a reduction in bone strength. In the available literature, no studies are showing the effect of nesfatin-1 on bone strength in conditions of established osteopenia. Thus, this paper is aimed at evaluating of the effect of nesfatin-1 on rat bone properties measured under the quasi-static and impact mechanical loading conditions. The use of the impact test to assess the mechanical properties of bones is a new aspect and may be of practical importance in the analysis of bone fracture resistance under natural loading conditions.

## 2. Materials and Methods

All experimental procedures on rats were carried out with the permission of the Local Ethics Committee at the University of Life Sciences in Lublin (23/2015) on 28 April 2015. The study was conducted on 21 female Wistar rats at the age of three months and with 210–230 g initial body weight (BW). The animals were exposed to a 12 h light and 12 h dark cycle at 22 °C ± 2 °C room temperature. The animals had access to standard laboratory chow (LSM, Agropol, Motycz, Poland) and water.

**Figure 1:**
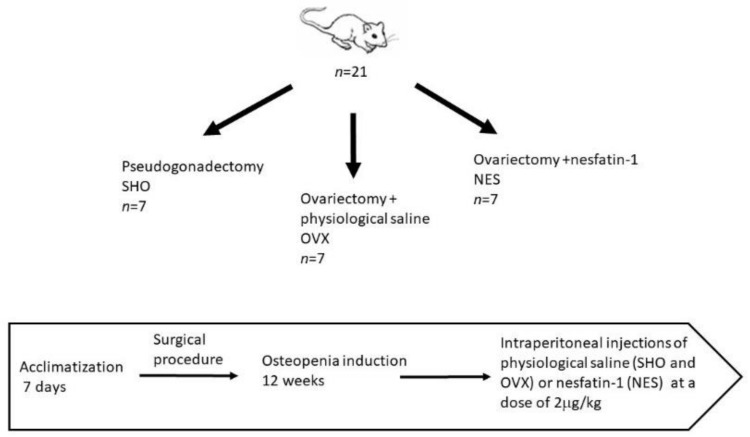
Experimental setup.

The animals were randomly divided into three experimental groups. The first control (SHO, *n* = 7) included rats in which pseudogonadectomy surgery was performed. Fourteen rats underwent bilateral removal of the ovaries. The procedures were performed under general anesthesia. In this case, intramuscular injections were used applying ketamine (Biowet Puławy, Puławy, Poland) in the dose of 3 mg/kg BW, xylazine (Biowet Puławy, Poland) in the dose of 10 mg, and atropinum sulphuricum (Polfa-Warszawa S.A., Warsaw, Poland) in the dose of 0.05 mg/kg BW. During the ovariectomy, after preparation of the operating field, a 7–8 cm incision of the abdominal wall was performed in the midline. The left and right ovaries were isolated from the uterine horns and then ligated and removed. Then, the operating wound was sutured. During the pseudogonadectomy, after incision of the abdominal wall, the viscera was manually repositioned, and then the operating wound was sutured. After surgery, the animals were kept under standard zoohygienic vivarium conditions for 12 weeks to induce osteopenia in the ovariectomized animals. After this period, the ovariectomized rats were randomized into two groups of 7 animals each. The OVX group received physiological saline by intraperitoneal injection. Rats in the NES group were administered nesfatin-1 at a dose of 2 µg/kg body weight (Phoenix Pharmaceuticals, Inc., Burlingame, CA, USA). The injections were made once daily for 8 weeks. Nesfatin was dissolved in saline before use. The rats in the SHO and OVX groups were administered saline in an amount corresponding to the nesfatin solution in the NES group. After 8 weeks, the animals were sacrificed by CO_2_ overdose preceded by 12 h night fasting (with water access). The scheme of the experimental setup is presented in Figure 1. After euthanasia, the isolated right and left humerus were subjected to densitometric measurements. Subsequently, the bones were secured at −20 °C for further mechanical analyses.

### 2.1. Densitometric Analysis (DXA)

Bone mineral density (BMD) and bone mineral content (BMC) were determined using the dual-energy X-ray absorption method (DXA). The study was performed on the right and left isolated humerus, previously cleared of soft tissues, using the Norland Excell Plus (Fort Atkinson, WI, USA) apparatus equipped with the Small Animal Scan 4.4.1 software. Whole bone scans were performed with the following parameters: initial scan 100 mm/s, resolution 1.5 mm *×* 1.5 mm, measure scan 30 mm/s, resolution 1.0 mm × 1.0 mm. Region of interest (ROI) was determined manually by the operator based on the initial scan. Before each measurement session, the densitometer was calibrated using a hydroxyapatite phantom provided by the manufacturer.

### 2.2. Quasi-Static Mechanical Analysis

After the densitometric analysis, the left humerus was subjected to the 3-point bending test according to the methodology presented in the work of Feretti et al. [29]. The analysis was used to evaluate the mechanical properties of the cortical bone, which usually corresponds to the mid-diaphysis of the bone. The three-point bending test was performed at room temperature on a ZwickRoell Testing Machine Z010 (ZwickRoell GmbH & Co. KG, Ulm, Germany). In the tests, the XForce HD head was used that enabled measurement in the range of maximum loads up to 10 kN, which moved at a constant speed of 10 mm/min. Each bone was placed horizontally on two supports with the anterior surface upward. Simultaneously, bone was evenly distributed on the supports, and the analyzed part of the bone was 40% of its length. From the load and displacement data, the following parameters were determined: ultimate strength as a maximal force registered under quasi-static loading condition (F_qs max_) and work-to-fracture (W_qs_). W_qs_ is defined as the area under the load–displacement curve and represents the work needed to fracture the bone. The length and diameter of the examined bones were similar. The differences did not exceed 5%.

### 2.3. Impact Study

**Figure 2:**
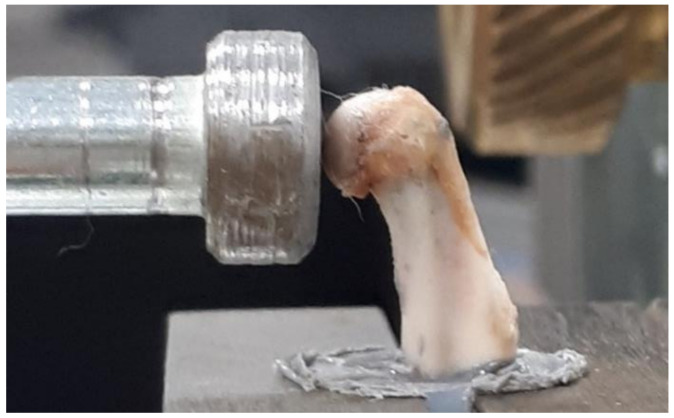
The position of bone and the hammer during the impact test.

The device for the impact study was composed of a base with two columns as load-bearing elements and three horizontal links. A pendulum with a rigid 924 mm long arm was mounted on the upper link. The arm was fitted with the measurement head equipped with a piezoelectric force sensor (2311-100 model, Endevco Corporation, Irvine, CA, USA) of 2.25 mV·N^−1^ sensitivity and a measurement range of ±220 N. The measuring head can be displaced in the vertical and horizontal plane, and its position was set by adjusting screws. The pendulum was connected with the angular displacement sensor RON 275 (Heidenhain, Traunreut, Germany) with the accuracy of 0.005°. The sensor recording inclination angle change was connected to the measuring card (SBC-68, National Instruments, Warsaw, Poland), transmitting signals to the application working in the LabView environment [9,10,11,30]. The right humerus was fixed with quick-drying epoxy glue in a PVC tube with an internal diameter of 15 mm and a 20 mm height. The bone was fixed vertically so that a deltoid tuberosity was above the glue surface. The sample was left in the air for 20 min to set the adhesive. After this time, the bone was placed in a specially designed holder. The humerus head was impacted at a velocity of 0.5m·s^−1^, which corresponded to the pendulum deflection by an angle of 9.15°. The position of the bone and the hammer during the impact test is presented in Figure 2.

**Figure 3:**
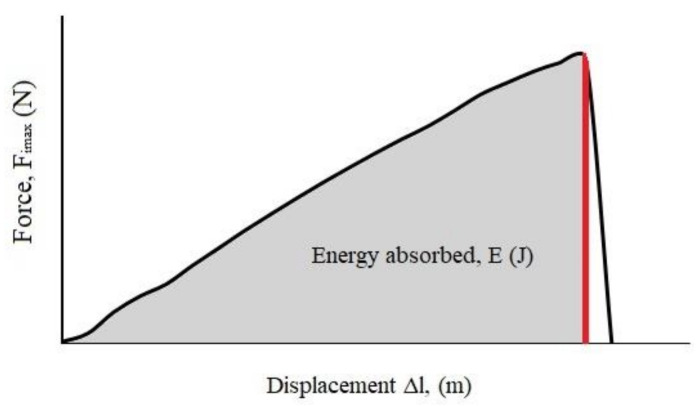
Calculation of absorbed energy from the force–displacement curve.

The obtained force–displacement curves were analyzed to determine the maximum force (fracture load) and energy absorbed during the impact (*E*). The energy was found from the area under the loading curve (Figure 3) by integrating to the maximum force (Graph, free software, version 4.4.2). It could also be determined from Equation (1), assuming that the kinetic energy of the hammer is many times larger at the time of impact compared to the energy required for the sample fracture [9]:

(1) $$E=v\int_{0}^{t}\left[F\left(t\right)\right]\;dt$$

where *v*—the velocity of the hammer at the impact time, assumed to be constant (m·s**^−^**^1^); *F(t)*—the force registered during the test as a function of time (N); *t*—the time from the beginning of the test to the point where the maximal impact force value was reached.

### 2.4. Fracture Surface Observations

Images showing the fracture surfaces of bones after the impact tests were obtained using the Nikon SMZ18 stereoscopic microscope equipped with a DS-Fi3 digital camera (Nikon Corporation, Tokyo, Japan) and the Phenom ProX scanning electron microscope (Thermo Fisher Scientific Inc., Waltham, MA, USA). The samples were placed on a standard aluminum slide with carbon adhesive. The bones were imaged without pretreatment with the accelerating voltage of 10 kV using the backscattering electron detector. The qualitative elemental distribution analysis was made using energy dispersive spectroscopy (EDS system) at a voltage of 15 kV.

### 2.5. Statistical Analysis

Statistical data analysis was performed in the STATISTICA 13.1 (StatSoft, Inc., Tulsa, OK, USA). The obtained results were presented as mean values with the standard deviation (mean ± S.D.). The one-way analysis of variance ANOVA with the Tukey post-hoc test was applied to test differences between the means. The significance level was evaluated at α = 0.05, and the differentiating factor was the rats’ treatment. The correlation between the maximum force obtained under the quasi-static (F_qs max_) and impact (F_i max_) loading conditions was analyzed by means of the Pearson’s correlation coefficient analysis.

## 3. Results and Discussion

### 3.1. Densitometric Analysis

The results of the densitometric analysis of the isolated bones are presented in Table 1. Left bone DXA results were comparable (results not shown). The highest mean value of BMD and BMC was observed for the control group. Ovariectomy caused a reduction of BMD by 9.37% and 4.63% in the case of OVX and NES, respectively, compared to the SHO group. The humerus BMC was also the largest for the control group and decreased by 8.79% for OVX and 3.94% for NES (Table 1). The authors’ findings are consistent with those obtained by other researchers [20,31,32]. The study indicates that removing endocrine ovarian function following ovariectomy led to a reduction in the values of BMD and BMC (Table 1). Loss of estrogen increases bone remodeling in ovariectomized rats, with a predominance of bone resorption processes over bone formation. Compared with the control group, higher resorption and formation activities were observed in the OVX animals [33,34]. The injection of nesfatin-1 had a beneficial effect on BMD and BMC parameters. The densitometric parameters of the humerus in the NES group were higher than in the OVX animals receiving physiological saline. However, according to performed one-way ANOVA test, the obtained differences between the BMD and BMC mean values were not statistically significant (at the level of significance α = 0.05).

### 3.2. Quasi-Static Loading Condition

The smallest ultimate strength (F_qs max_) obtained under the quasi-static loading condition was observed for the OVX group (Table 2). The mean value of F_qs max_ for this group was smaller by about 15% than the control group. The greatest mean value of F_qs max_ was found in the case of the NES group. The statistical analysis showed that the mean values of these two groups were statistically different (*F*-value = 3.91, *p* < 0.05). In the SHO and NES rats, the mechanical parameters obtained in the three-point bending test were similar. In the case of work-to-fracture (W_qs_), the highest value was obtained in the sham-operated rats. Ovariectomy caused a decrease in this parameter by 34%, but the observed differences in W_qs_ between the analyzed humerus groups were not statistically significant (Table 2).

The results of the three-point bending test of the bones confirm those of the densitometric analysis predicting the mechanical strength. Other authors also indicated that gonadectomy caused a decrease in the mechanical bone parameters under the quasi-static loading conditions [35]. The observed increase in the F_qs max_ and W_qs_ indicate that nesfatin-1 reduced the negative effects caused by estrogen deficiency. The differences in the maximum force values between the OVX and NES humerus were statistically significant. In rats receiving nesfatin-1, F_qs max_ values were similar to those observed for the control group (Table 2).

### 3.3. Impact Loading Conditions

**Figure 4:**
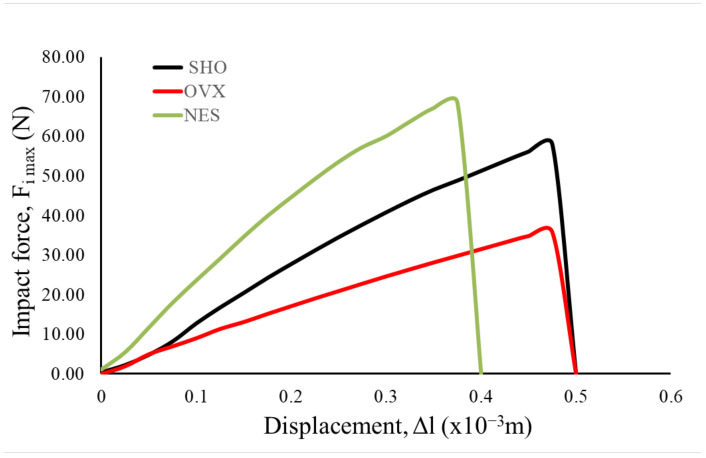
Typical courses of sample response recorded by the sensor installed in the hammer during impact.

The mechanical analysis results under the impact loading conditions confirm the results of the three-point bending test carried out under the quasi-static conditions. It was proved that ovariectomy leads to maximum force (F_i max_) decrease, registered in the impact test. The average value of F_i max_ was 36.23 N (Table 3). For this group, the lowest slope of the force–displacement curves was also observed (Figure 4).

The samples of the NES group were characterized by the highest values of F_i max_ (Figure 4, Table 3). For this group, the highest slope of the force–time curves was also observed. The one-way ANOVA test showed that the differences between the maximal force mean values of all analyzed groups were statistically significant at the level of significance α = 0.05 (F-value = 115.57, *p* < 0.05). These results showed that the impact test was more sensitive than the classical quasi-static three-point bending test.

In the case of energy (E) absorbed during impact (Table 2), the highest mean value was observed for the SHO group (16.35 × 10^−3^ J). The lowest mean value of absorbed energy was obtained for the OVX group. The one-way ANOVA test showed that the E mean values between these two groups are statistically different at the level of significance α = 0.05 (F-value = 12.39, *p* < 0.05). An increase in the mean energy was observed for the humerus from the NES group to the OVX group. However, the E mean value was lower than that for the SHO group. Analyzing the linear displacement values, it can be seen that the smallest values of ∆l were obtained in the case of the rat group receiving nesfatin-1, whereas the largest value of displacement was observed for the sham-operated group. As the performed analysis of variance showed, the obtained differences were not statistically significant (Table 3). However, this observation could indicate that nesfatin-1 increased the mechanical strength and, at the same time, the bone could become more fragile.

The significance of nesfatin-1 in bone physiology and characteristics is still little-known. It was found that the administration of nesfatin-1 leads to an increase in bone densitometric parameters in ovariectomized animals [16]. Other authors [36] showed that the application of NUCB2/nesfatin-1 in female rats after gonadectomy had a positive effect on immunohistochemical reaction in all zones of the growth cartilage of long bones. The growth plate thickness in rats receiving nesfatin was similar to that in animals subjected to the sham operation. An increase in the level of nesfatin-1 was also observed in the rats’ blood after gastrectomy. This has been associated with osteopenic changes following the surgery [37]. These findings, together with our results, suggest that changes in the level of nesfatin-1 may have a positive protective effect on bone metabolism.

**Figure 5:**
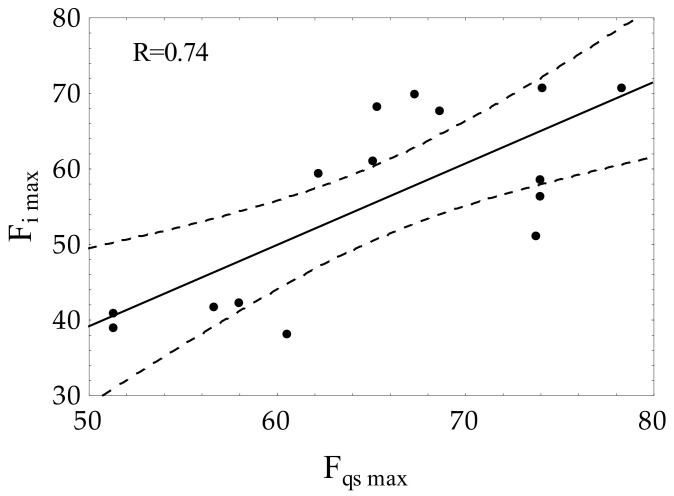
Correlation between the bone strength parameters obtained under the quasi-static (F_qs max_) and impact loading (F_i max_) conditions.

The correlation analysis (Figure 5) showed a positive Pearson’s correlation coefficient (R = 0.74) between the maximum forces measured in the impact test (F_i max_) and under the quasi-static condition (F_qs max_). The above indicates that values of F_i max_ can be used for the evaluation of bone toughness together with other mechanical parameters.

### 3.4. Fracture Surface Observations

**Figure 6:**
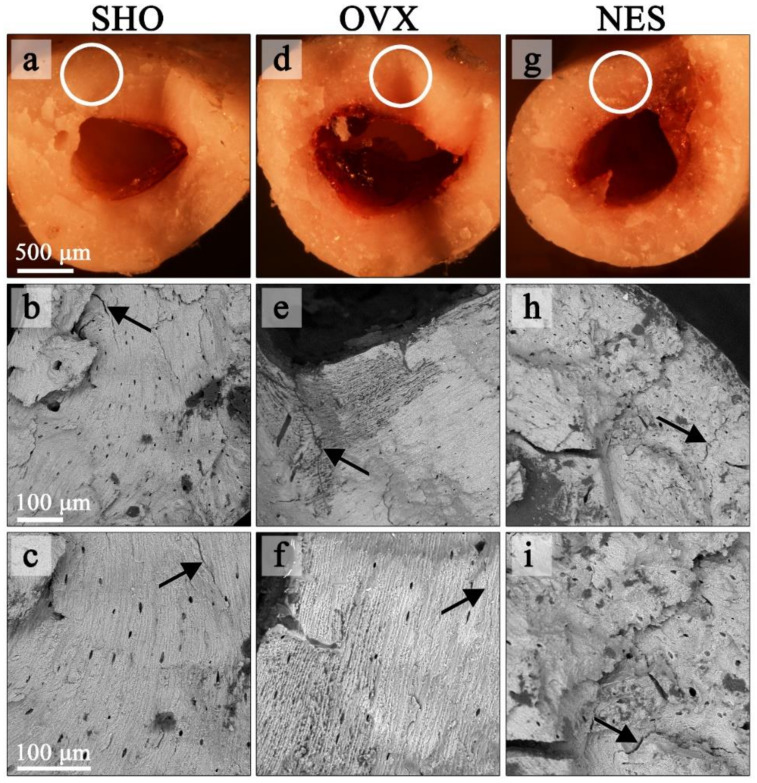
Stereoscopic (**a**,**d**,**g**) and SEM images (**b**,**c**,**e**,**f**,**h**,**i**) of bone fracture surfaces in the SHO, OVX, and NES groups. The SEM-imaged areas are marked with a circle on the stereoscopic photos. Arrows show bone tissue cracks.

**Figure 7:**
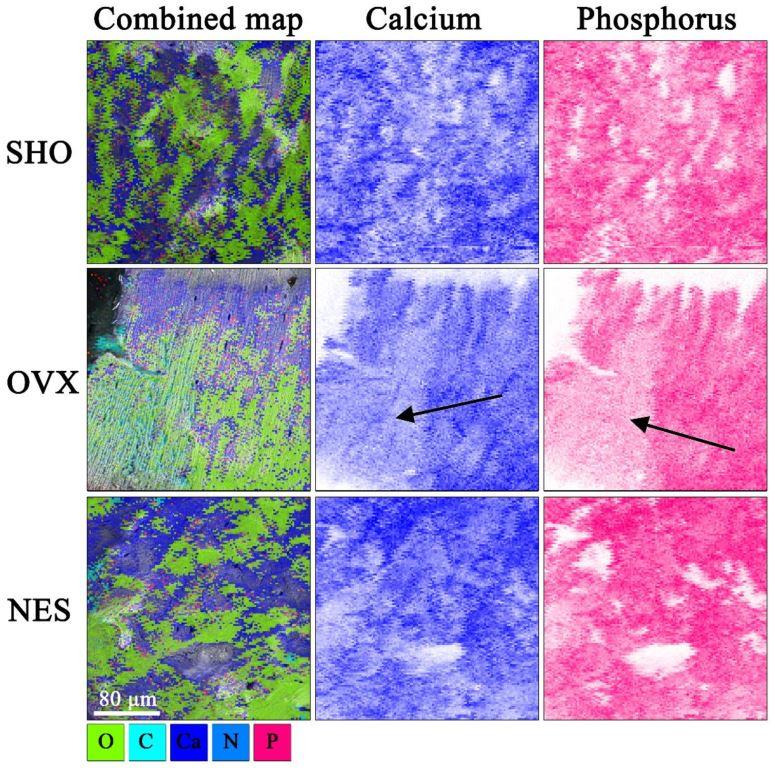
SEM–EDS elemental mapping. The black arrows show the areas with reduced calcium and phosphorus content in OVX humerus.

Figure 6 demonstrates the structural differences between the bones from the control, ovariectomized, and ovariectomized receiving nesfatin-1 rat groups. The cracks in the SHO bones traveled along the lamellae and propagated straight through the overall bone structures (Figure 6a–c). A straight crack path and a smooth fracture surface could be a consequence of both the dynamic loading and porous bone microstructure. A straight crack path and a smooth fracture surface were also observed in the OVX group (Figure 6d–f). However, the SEM images of the humerus fracture surface in this group showed that ovariectomized rats had a much looser bone structure in some areas with many micropores, indicating a bone mineral loss. Our findings are in line with studies of other authors. Bhardwaj et al. noticed lost connectivity of trabeculae following the rat’s ovariectomy [38]. Simultaneously, the authors found a calcium content decrease in the osteopenic rat’s femur. In EDS maps (Figure 7), the looser bone structure is indicated with a black arrow. Qualitative EDS analysis showed a Ca content decrease in these areas (Figure 7), which affects the bone mechanical strength. The surrounding bone tissue clearly shows a higher content of calcium and phosphorus. These observations are consistent with the densitometric analysis (Table 1). The effect of estrogen deficiency on changes in bone structure is not uniform [39]. Liu et al. [40] observed that 4 weeks postovariectomy, bone losses in rats were significant. The response to dynamic loading is different for cortical and cancellous bones. Cortical bone is stiffer and can withstand higher stress but lower strain. Trabecular bone, as a porous material, is characterized by elasticity associated with resistance to lower stress and higher strain [41,42]. Moreover, the strength and fracture toughness of the cortical bone depends on its location. Long bones like femora and humeri show a higher thickness of the cortical shell in the diaphyseal areas than the metaphyseal ones [41,42,43].

Cracks of rat bones receiving nesfatin-1 took a highly zig-zag path [4]. There were a large number of crack deflections in a short distance (Figure 6g–i). Such a crack path could be due to the greater rigidity of bones in this rat group. Nesfatin-1 is derived from NUCB-2, which can attach Ca^2+^. Peterson et al. [44] reported that NUCB exhibited intra- and extracellular localization within the bone tissue. Puzio et al. [14] obtained similar results for cartilage of the distal metaphysis of the rat femur. The authors suggested that nesfatin-1 can act as a modulator of bone matrix maturation and possibly be of importance in Ca^2+^ transport during bone formation [14,44]. The EDS qualitative analysis showed an increase in Ca^2+^ content in the NES samples in relation to the OVX group, which could be related to an increase in the bone strength.

## 4. Conclusions

The experiment showed that bone strength parameters measured under various mechanical loading conditions increased after the nesfatin-1 administration. Critical energy (energy to the fracture) values were similar for the control and animals receiving nesfatin-1; however, a larger slope of the force–displacement curve suggests an increase in bone stiffness in the rats after the nesfatin-1 treatment. The bones of the ovariectomized rats were most susceptible to deformation and had the smallest fracture toughness. The impact test was more sensitive compared to the classical quasi-static three-point bending test. Statistically significant differences were observed between the mean F_imax_ values of all analyzed sample groups. F_qs max_ differentiated only the ovariectomized group from the control and the nesfatin-1-receiving rat females. The results allow concluding that F_i max_ could be used as a parameter to predict bone fracture toughness. To obtain complete characteristics of mechanical bone parameters under the impact loading conditions, further analysis should be conducted using a larger impact velocity. This could provide more information about bone fracture toughness.

## Figures and Tables

**Table 1:** Densitometric parameters of isolated right rat humerus in the developing osteopenia condition. BMD—the mineral density ± S.D.; BMC—the mineral content ± S.D.

Group	BMD (g/cm^2^)	BMC (g)
SHO	0.0928 ± 0.0052	0.1854 ± 0.0150
OVX	0.0841 ± 0.0066	0.1691 ± 0.0178
NES	0.0885 ± 0.0028	0.1781 ± 0.0072

**Table 2:** Mechanical parameters obtained under the quasi-static loading conditions. The same letter means no significant differences between the values at the level of significance α = 0.05, one-way ANOVA variance analysis, Tukey’s HSD test.

Group	F_qs max_(N)	W_qs_ (N·mm)
SHO	69.85 ± 5.71 ^a,b^	13.38 ± 3.31
OVX	55.56 ± 4.16 ^a^	8.81 ± 2.33
NES	70.73 ± 5.35 ^b^	11.76 ± 4.12

**Table 3:** Mean values with the standard deviations of maximal force (F_i_), energy absorbed during impact (E), and linear displacement (Δl). The same letter means no significant differences between the values at the level of significance α = 0.05, one-way ANOVA variance analysis, Tukey’s HSD test.

Group	Maximum Impact Force, F_i max_ (N)	Energy, E (J × 10^−3^)	Linear Displacement, Δl (m × 10^−3^)
SHO	57.11 ± 3.88 ^a^	16.55± 2.08 ^a^	0.65 ± 0.17 ^a^
OVX	40.23 ± 2.82 ^b^	10.36 ± 2.53^a^	0.56 ± 0.08 ^a^
NES	69.35 ± 3.40 ^c^	17.44 ± 7.01^a^	0.46 ± 0.14 ^a^

## Data Availability

Data is contained within the article.
